# The Association of Thyroid Disease and Oral Lichen Planus: A Literature Review and Meta-analysis

**DOI:** 10.3389/fendo.2017.00310

**Published:** 2017-11-09

**Authors:** Dan Li, Jin Li, Chunlei Li, Qianming Chen, Hong Hua

**Affiliations:** ^1^Department of Oral Medicine, Peking University Hospital of Stomatology, Beijing, China; ^2^Department of Oral Medicine West China School of Stomatology, Sichuan University, Chengdu, China

**Keywords:** thyroid disease, oral lichen planus, hypothyroidism, hashimoto thyroiditis, pathogenesis, meta-analysis

## Abstract

**Background:**

The objective of this study was to systemically evaluate the association between oral lichen planus (OLP) and thyroid disease.

**Method:**

Eleven electronic databases were searched for any clinical study published up until August 2016 that explored an association between OLP and thyroid disease, complemented with manual searching. The titles and abstracts of all studies identified from the electronic searches were then assessed independently to determine inclusion of the study. The population of interest was patients with OLP, and the exposure was the presence of thyroid disease in OLP patients. Statistical analyses were conducted using Review Manager 5.2. The odds ratios (OR) and 95% confidence intervals (CIs) were calculated to evaluate the association between OLP and thyroid disease.

**Results:**

Eight studies were included for review, and of these, four case-control studies were included in the final meta-analysis. The mean Newcastle–Ottawa Scale score evaluating the methodological quality of the four studies was 6.5. The OR was 2.10 (95% CI: 1.47–3.01), indicating a statistically significant difference in the prevalence of thyroid disease between the OLP and control groups. Heterogeneity was satisfactory (*I*^2^ = 0%, <25%). An approximately symmetrical funnel plot suggested no obvious publication bias. Two articles showed a higher correlation between OLP and hypothyroidism (OR 1.83, 95% CI: 1.16–2.89), with satisfactory heterogeneity (*I*^2^ = 0%, <25%).

**Conclusion:**

This meta-analysis indicated a significantly high prevalence of thyroid disease among OLP patients compared with controls, suggesting that routine screening for thyroid disease could be beneficial to OLP patients. However, due to the small number of studies included, further studies are needed to confirm the results.

## Introduction

Oral lichen planus (OLP) is a chronic inflammatory mucocutaneous disease most commonly affecting middle-aged females. It is estimated to affect 0.5–2.0% of the general population (1–3). Some researchers believe a complex series of immune-modulated events is responsible for the pathogenesis of OLP (4); however, the factor that initially triggers OLP remains unknown. The few predisposing factors currently thought to have a role in its pathogenesis include the patient’s genetic background (5) and psychological factors such as periods of psychological stress and anxiety (6–8). In some populations, OLP has been reported to be associated with certain systemic diseases, including hepatitis C virus, hypertension, diabetes mellitus, and thyroid disease (2, 3, 9, 10).

The thyroid is a secretory organ secreting thyroid hormones, which control many aspects of growth, development, regeneration, and metabolism. Deviations from normal thyroid hormone levels in either direction occur quite frequently and are very unpleasant for patients (11). The most common thyroid disorders include thyroid nodules, hyperthyroidism, autoimmune thyroid diseases (AITDs) caused by abnormal autoantibodies, and so on. The human AITDs broadly include Graves’ disease and Hashimoto’s thyroiditis (HT), which are the most common causes of thyroid gland dysfunction and nonendemic goiter (12). HT, the most common cause of hypothyroidism, involves antibodies that directly bind to thyroid antigens (13). Graves’ disease, on the other hand, involves the binding of autoantibodies to the TSH receptor, which leads to stimulation, and is the most common cause of thyrotoxicosis (14). In 2012, a review summarized studies of autoimmune thyroid disease incidence and prevalence since 1950, found high prevalence and incidence of hypothyroidism and prevalence for TPO antibodies in Denmark, Finland, Norway, and other European countries (15). Hypothyroidism and hyperthyroidism affect renal function by direct renal effects as well as systemic hemodynamic, metabolic, and cardiovascular effects (16). However, the pathogenesis of AITDs remains unclear.

A correlation between thyroid disease and OLP was first identified in a case report in 1994 (17), and several studies exploring this correlation have been conducted since. One study found that OLP patients have a higher prevalence of thyroid disease, especially hypothyroidism, compared with controls (18). In 2009, a study conducted in Taiwan by Sun et al. found that 21.3 and 24.4% of Chinese OLP patients had significantly higher levels of serum antithyroglobulin autoantibodies and antithyroid microsomal autoantibodies, respectively, compared with healthy controls (19). Another study published in 2013 indicated a higher prevalence of HT in an OLP group relative to the general population (20). However, this relationship between OLP and thyroid disease still remains controversial. The aim of this study was to systemically analyze the relationship between OLP and thyroid disease.

## Methods

This review was performed following the preferred reporting items for systematic reviews and meta-analyses (PRISMA) guidelines (21).

### Criteria for Selection

Articles based on clinical studies that explored the relationship between OLP and thyroid disease were considered for inclusion. Any type of study was considered, including case-control, cross-sectional, and cohort studies, either with or without a control group. Studies involving patients clinically diagnosed with OLP, with or without a pathological diagnosis, were included. No restrictions on age, sex, race, or country were set. The exposure of interest was the presence of thyroid disease, and the main outcome indicator was an odds ratio (OR) for OLP and thyroid disease. Studies including a control group were identified, so that their data could be merged to calculate the ORs and 95% confidence intervals (CIs). Studies that did not include a control group were retained for literature review.

### Search Strategy

Studies in English or Chinese that met the above criteria were collected by searching the following databases: PubMed, MEDLINE, Cochrane Library, Science Direct, Web of Science, Embase, China National Knowledge Infrastructure, China Science and Technology Journal Database (CQVIP), Wanfang Data, Chinese Biomedical Literature Database, and the Chinese Science Citation Database. The MeSH terms (“oral lichen planus” OR “lichen planus” OR “OLP” OR “LP”) AND (“thyroid disease” OR “hypothyroidism” OR “thyroid nodule” OR “hyperthyroidism” OR “Hashimoto disease” OR “Hashimoto’s thyroiditis” OR “thyroiditis”) were searched, complemented with manual searching, for publications listed up until August 2016. The searches were conducted by two independent investigators (Dan Li and Jin Li).

### Study Selection

Two of the authors independently assessed the titles and abstracts of all identified studies. Full reports were obtained for those studies that appeared to meet the inclusion criteria or those for which a clear decision could not be made from the title and abstract alone. Where disagreements occurred, they were resolved by discussion or by consulting a third author (Chunlei Li and Hong Hua).

### Data Extraction and Management

Data extraction and management were performed independently by two reviewers (Dan Li and Jin Li). The following data were recorded from each study: authors, country of origin, study type, the main outcome measures, and the outcomes.

### Statistical Analysis

Statistical analyses were conducted using Review Manager 5.2 (Cochrane Collaboration). The estimates of the association between OLP and thyroid disease were expressed as ORs and 95% CIs and plotted on a forest plot. The *I*^2^ test was performed to evaluate the heterogeneity among the studies, with possible values of 0–100%; *I*^2^ values of 25 and 50% were used as cutoffs for modest and high heterogeneity (22). If no significant heterogeneity was found, a fixed-effects model was used to calculate the combined OR. Otherwise, a random-effects model was used.

### Risk of Bias

The methodological quality of the included studies was assessed using the Newcastle–Ottawa Scale for the case-control studies (23). Funnel plots were used to evaluate underlying publication bias, and a sensitivity analysis was performed to identify the influence of the individual studies on the combined OR.

## Results

### Study Selection and Characteristics

**Figure 1:**
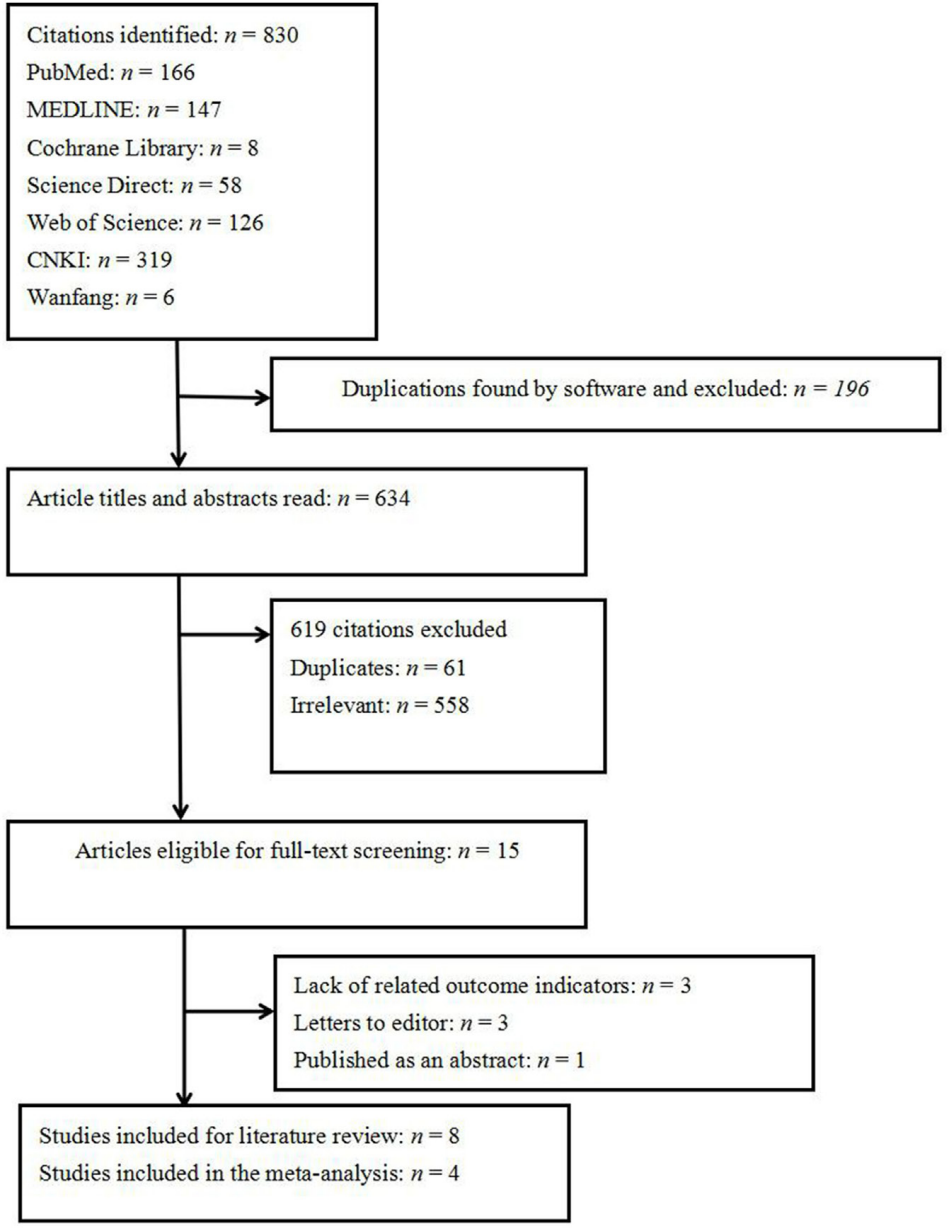
Systematic review flow diagram.

A total of 830 papers were initially identified. Of these, 765 citations were excluded because they were duplicates, had irrelevant contents, or did not meet the inclusion criteria. Of the 15 eligible full-text studies, 3 were excluded because of a lack of related outcome indicators and outcomes, 1 abstract published in a supplemental journal of oral disease was excluded because of no full text (24), and 3 letters to journal editors were also excluded (Figure 1). Finally, eight studies were eligible for inclusion (Table 1). These studies covered a broad geographical range, with five conducted in Europe, two in Asia, and one in both Oceania and Europe. Four of the eight included studies did not feature a control group, and thus the OR could not be estimated. The four remaining studies, with a total of 1,846 OLP patients, were included in the meta-analysis.

**Table 1:** Characteristics of the included studies for association of oral lichen planus and TD.

Study	Country	Study type	Study group (oral lichen planus)		Control group		OR (95% CI)

			Total	TD	Total	TD	
Lavaee and Majd (25)	Iran	Retrospective comparative study	523	35 (6.7%)^b^	523	21 (4%)^b^	1.714 (0.984–2.987)
Garcia-Pola et al. (26)	Spain	Case-control study	215	33 (15.3%)	215	12 (5.6%)	3.0673 (1.537–6.117)
Robledo-Sierra et al. (27)	Sweden	Case-control study	956	59 (6.2%)	1,029	16 (1.6%)	a
Siponen et al. (18)	Finland	Case-control study	152	22 (15%)	222	18 (8%)	2.12 (1.06–4.21)
				15 (10%)^b^		11 (5%)^b^	2.39 (1.05–5.61)^b^
Muzio (20)	Italy	Cross-sectional study	105	15 (14.3%)^c^	^a^	(1%)^c^	^a^
Vanja (9)	Australia	Cross-sectional study	163	15 (9.2%)	^a^	^a^	^a^
	Croatia		160	22 (13.7%)			
Lauritano et al. (10)	Italy	Retrospective study	87	13 (15%)^d^	^a^	^a^	^a^
Munde et al. (28)	India	Retrospective study	128	1 (0.78%)^b^	^a^	^a^	^a^

*TD, thyroid diseases*.

*^a^Original data unavailable in the full text*.

*^b^Patients with hypothyroidism*.

*^c^Hashimoto thyroiditis*.

*^d^Thyroiditis*.

### Relationship between OLP and Thyroid Disorders

**Figure 2:**
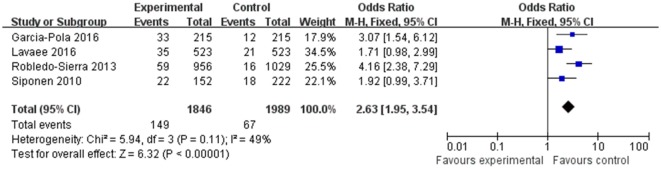
Heterogeneity of the included studies.

**Figure 3:**
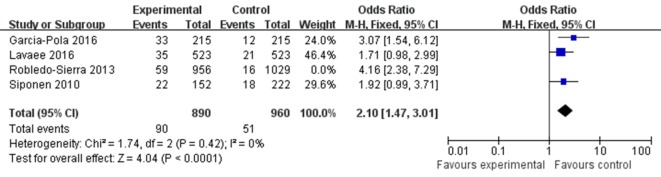
Sensitivity analysis of the included studies.

**Figure 4:**
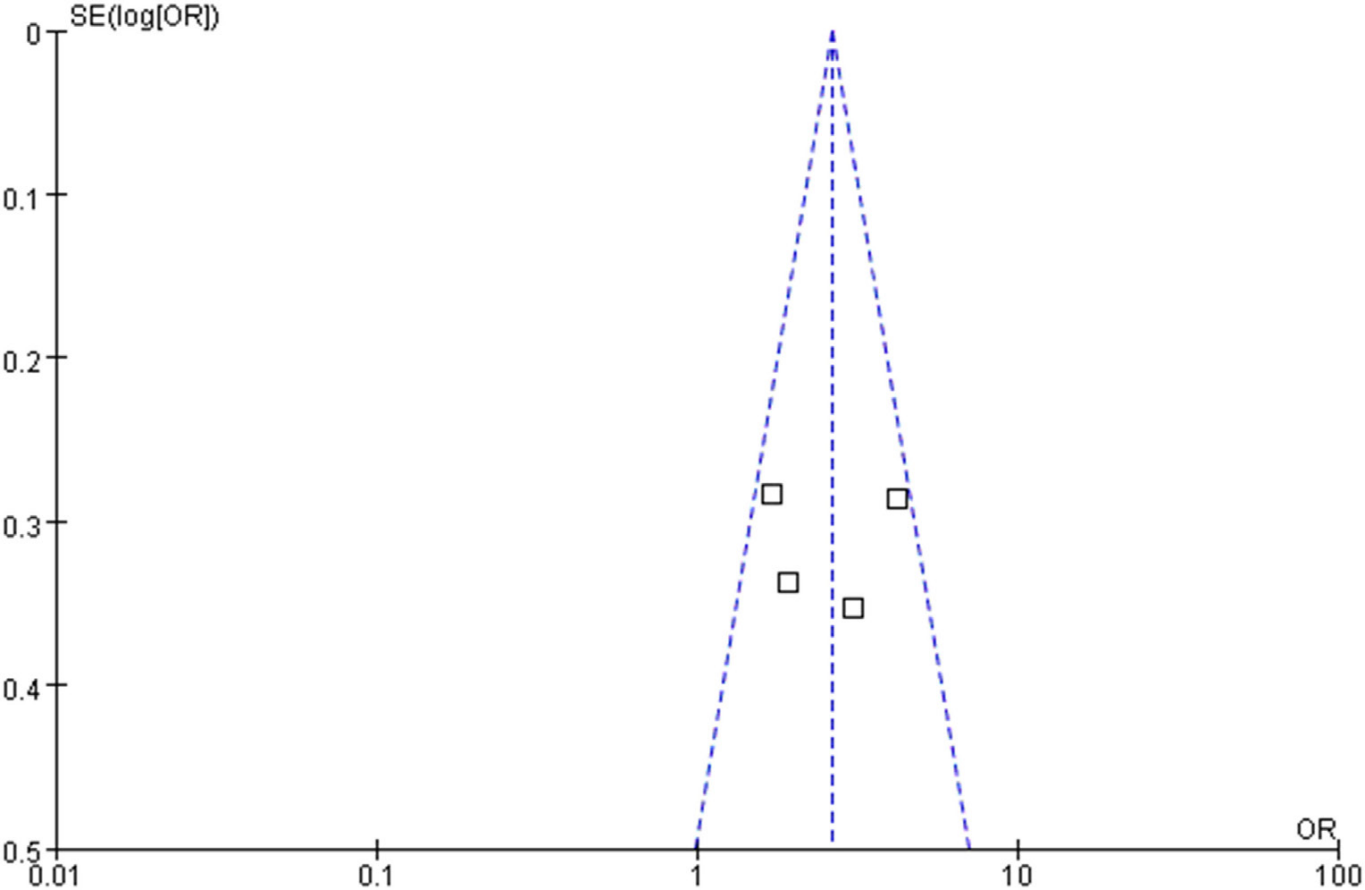
Funnel plot of the included studies.

The OR for the association between OLP and thyroid disease varied from 1.71 (95% CI: 0.98–2.99) to 4.16 (95% CI: 2.38–7.29), demonstrating a statistically significant difference in the prevalence of thyroid disease between OLP patients and controls. The heterogeneity among the included studies (*I*^2^) was 49% (>50%, Figure 2), representing critical heterogeneity; thus, a fixed-effect model was used for the meta-analysis. The pooled OR was 2.63 (95% CI: 1.95–3.54). Following the sensitivity analysis, heterogeneity was more satisfactory at *I*^2^ = 0% (<25%, Figure 3), with a combined OR among the three studies of 2.10 (95% CI: 1.47–3.01). The methodological quality of the four included studies was evaluated, with a mean score of 6.5 (Table 2). The funnel plot was approximately symmetrical, suggesting no obvious publication bias (Figure 4).

**Table 2:** Methodological quality of the included studies based on the Newcastle–Ottawa Scale for assessing the quality of case-control studies.

Study	Selections (score)				Comparability (score)	Exposure (score)			Total scores
	Adequate definition of patient cases	Representativeness of patient cases	Selection of controls	Definition of controls	Control for important factor or additional factor	Ascertainment of exposure	Same method of ascertainment for cases and controls	Non-response rate	
Lavaee and Majd (25)	1	1	1	1	1	0	0	1	6
Garcia-Pola et al. (26)	1	1	1	1	1	1	1	1	8
Robledo-Sierra et al. (27)	0	1	0	1	0	0	1	1	4
Siponen et al. (18)	1	1	1	1	1	1	1	1	8

### Relationship between OLP and Hypothyroidism

**Figure 5:**
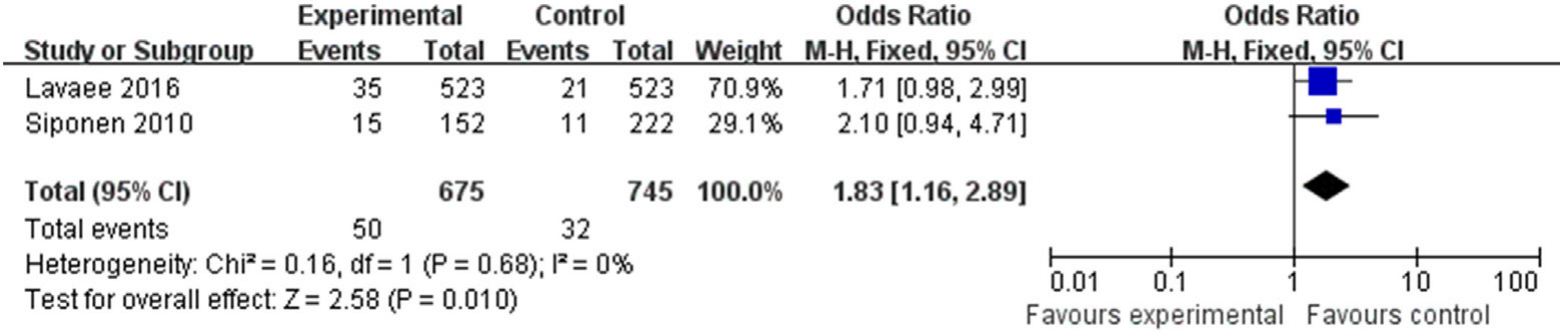
Heterogeneity of the studies on hypothyroidism.

Two of the eight articles provided data pertaining to hypothyroidism. The data were merged (Figure 5) and revealed a correlation between OLP and hypothyroidism (OR 1.83, 95% CI: 1.16–2.89), with satisfactory heterogeneity (*I*^2^ = 0%, <25%).

## Discussion

Oral lichen planus is recognized as an autoimmune mucocutaneous disease and is more common among females. Multiple factors have been identified as potentially contributing to its pathogenesis. The prevailing theory is that a complex series of immune-modulated events is responsible for OLP pathogenesis, but the main cause of OLP remains unknown. In recent years, the association between OLP and thyroid disorders has drawn attention. A study from 2010 showed a correlation between OLP and thyroid disease in a Finnish population (OR 2.12, 95% CI: 1.06–4.21) (18). There are other reports linking OLP and thyroid disease (25–28), but until now, the correlation between OLP and thyroid diseases has been questionable. Hence, we performed this review and meta-analysis.

We ultimately included eight articles that met the inclusion criteria, and data from four of these eight were combined for meta-analysis. Following a sensitivity analysis, we found that patients with OLP were significantly more likely to have thyroid disease than controls (OR 2.10, 95% CI: 1.47–3.01). There was no obvious publication bias, and three of the four studies had a high methodological quality score. Heterogeneity was satisfactory at *I*^2^ = 0%, after excluding the data from the study by Robledo-Sierra et al. (27), which had relatively low methodological quality. The results lead us to conclude that there is a strong link between OLP and the presence of thyroid disease, consistent with most previous studies.

Hypothyroidism and HT were the most frequently identified thyroid diseases in the studies. A possible link between HT and OLP was reported in an article by Lo et al. in 2013, who reported a prevalence of HT of 14.3% in a group of patients with OLP. The prevalence of HT-related hypothyroidism in the general population was 1%; thus, the difference was statistically significant (OR 14.29, 95% CI: 1.9–16.2; *P* < 0.0003) (20). Two of the eight articles provided data on hypothyroidism alone, and the combined data demonstrated a correlation between OLP and hypothyroidism (OR 1.83, 95% CI: 1.16–2.89). This is in contrast to the finding of Lavaee and Majd, with result showed no significant association between hypothyroidism and OLP (OR 1.714, 95% CI: 0.984–2.987) (25), and further research is needed to validate the results.

The causes of OLP and HT-related hypothyroidism are similar. OLP is considered to be a T-cell-mediated autoimmune disease in which auto-cytotoxic CD8 + T cells trigger apoptosis of the basal cells of the oral epithelium (2). HT is an organ-specific autoimmune disease, and its pathogenesis involves release of perforin by CD8+ cytotoxic T cells, and particle enzymes are considered the main cause of thyroid cell damage, eventually leading to hypothyroidism (29, 30). Both OLP and thyroid disease are more common in women than in men according to previous reports. In 2008, age-standardized prevalences of 1.27% overall, 0.96% in men, and 1.57% in women were calculated from a review article that extracted data from valid studies reporting the prevalence of OLP (31). A retrospective study evaluated the incidence of OLP in southeast Serbia over a 10-year period; women were found to be significantly more likely to have OLP (*P* < 0.001) (32). In the OLP groups of the four case-control studies included in our analysis, 65.4–74% were women, consistent with previous reports. A cohort study published in 1995 reported a higher incidence of thyroid disease in women than in men in a 20-year follow-up survey (12).

The Mechanism between OLP and thyroid diseases need to be further elucidated. From this study, this association between OLP and HT-related hypothyroidism suggests that thyroid disease may be involved in the pathogenesis of OLP. A molecular mimicry hypothesis was proposed for both lichen planus and HT, in which structural similarities between microbial antigens and human autoimmune reactions can turn a defensive immune reaction into an autoimmune reaction (33). However, the thyroid diseases in the articles analyzed in this study were not confined to hypothyroidism and HT. We suggest that OLP patients, especially women, should undergo routine screening for thyroid disease. Serological levels of thyroid stimulating hormone, T3 and T4, are indicators of thyroid function, and antithyroid peroxidase autoantibody and antithyroglobulin antibodies may be detected in AITDs. B-scan ultrasonography can be used to detect lesions such as thyroid nodules or as a secondary test for HT (34).

## Conclusion

The etiology of OLP is unclear; however, we found a positive and statistically significant correlation between OLP and thyroid disease. From this, we can assume that thyroid disease may be involved in the pathogenesis of OLP, or that OLP is a clinical manifestation of thyroid disease. To verify this conclusion, more reliable clinical studies are needed. Routine screening for thyroid disease as part of therapeutic intervention for OLP is suggested. This may also help elucidate the pathogenesis of, as well as identify new treatment strategies, for OLP.

## Author Contributions

HH and QC conceived and designed the research and critically revised the manuscript. DL and CL drafted the protocol. DL and JL participated in the search, screening, and analysis of the literature and in writing the manuscript.

## Conflict of Interest Statement

The authors declare that the research was conducted in the absence of any commercial or financial relationships that could be construed as a potential conflict of interest. The reviewer SF and handling editor declared their shared affiliation.
